# On the pressure wave emanating from a deflagration flame front

**DOI:** 10.1016/j.heliyon.2024.e26012

**Published:** 2024-02-07

**Authors:** V. Bisio, F. Montomoli, S. Rossin, V.L. Tagarielli

**Affiliations:** a Department of Aeronautics, Imperial College London, SW7 2AZ, UK; b Baker Hughes, Via Felice Matteucci 2, 50127, Firenze, FI, Italy

**Keywords:** Deflagration, Blast, Pressure wave, Hydrocarbon

## Abstract

We consider a spherical flame expanding from an ignition point through a homogeneous, flammable gaseous mixture. We analytically predict the transient pressure and velocity fields ahead of the flame as a function of the flame front position, which is assumed to evolve in time according to a power-law relation. The predictions are successfully validated by CFD simulations. We show that the model is also effective for analyzing real deflagration problems, by predicting measurements taken in hydrocarbon deflagration tests.

## 1 Introduction

The problem of accidental gas deflagration is of interest to the safety of most industrial operations, and the rapidly growing adoption of highly flammable and energetic hydrogen as a fuel has recently renewed the interest in deflagration problems [[1], [2], [3], [4], [5]]. In the majority of the reported accidental deflagration events, deflagrations occurred due to the ignition of accidental releases of flammable gas into confined or partially confined, often congested areas. Insufficient prevention and lack of mitigation measures led to uncontrolled escalation of events. Minimization of the likelihood of accidental deflagrations and of their consequences became therefore one of the first priorities in the field of energy industry safety. This research aims at providing a simple tool to aid the quantification of the consequences of gas deflagration. The published literature lacks fast and reliable prediction methods to aid designing against deflagration in industrial operations. While numerical simulations can capture deflagration events in detail, their cost can be computationally prohibitive and industrial engineers need analytical methods to analyse accidental deflagrations and their effects on surrounding structures.

In this paper we provide a simple practical method to evaluate the pressure wave radiated by a spherical deflagration, in the event of ignition of a flammable gas cloud in uncongested, unconfined environments.

We assume here that the flame front can be treated as a rigid and outwardly expanding spherical surface, as in early work by Taylor [6] and others [3,4]. Since the overpressures induced by unconfined spherical deflagration are typically of order of kPa, and therefore small compared to the initial atmospheric pressure, we further assume that the fluid is incompressible. Several authors [[7], [8], [9], [10]] have shown that the radius of the flame front R evolves in time according to a power-law of type $R=R_{0}+At^{B}$; here t represents time, $R_{0}$ is the effective size of the initial ignition region, and A and B are empirical parameters, which are tabulated in (e.g.) [7] for different types of gas mixtures, with B found often close to 1.5. In this study we also assume that the evolution of the flame front position follows this power-law.

### 1.1 Prediction of the pressure wave

Consider a spherical deflagration flame front at radial r=R(t) in a spherical reference system, advancing with time t as $R=R_{0}+At^{B}$. Following [6], the velocity potential of the flow has the form

(1) $$\Phi =\frac{1}{r}f\left(r-at\right)$$

where r is the radial position in a spherical coordinate system, a is the sound speed in the medium and f is a function to be determined. The radial velocity field u ahead of the flame, by definition of velocity potential has the form

(2) $$u=\frac{1}{r^{2}}f\left(r-at\right)-\frac{1}{r}f^{\prime }\left(r-at\right)$$

where the prime symbol denotes differentiation, and the pressure field p can be calculated as

(3) $$p-p_{0}=-\rho \frac{a}{r}f^{\prime }\left(r-at\right)$$

where $p_{0}$ is the initial (in this case atmospheric) pressure and ρ is the density of the fluid. Due to the expanding rigid surface assumption, at the flame front of radius R it must be

(4) $$\dot{R}=\frac{1}{R^{2}}f\left(R-at\right)-\frac{1}{R}f^{\prime }\left(R-at\right)$$

where the overdot denotes differentiation with respect to time. Taylor [6] notes that a closed-form solution of eq. (4) can be found for the case of constant $\dot{R}$ and $R_{0}=0$. In our case however the flame speed is not constant and this condition reads

(5) $$ABt^{B-1}=\frac{f\left(R-at\right)}{{\left(R_{0}+At^{B}\right)}^{2}}-\frac{f^{\prime }\left(R-at\right)}{R_{0}+At^{B}}$$

Now let $g\left(t\right)={R-at=R}_{0}+At^{B}-at$ and h(t)=f(g(t)). Note that $\dot{h}=f^{\prime }\left(R-at\right)\dot{g}$, such that eq. (5) can be written as

(6) $$\dot{h}\left(t\right)=h\left(t\right)\frac{\left(ABt^{B-1}-a\right)}{\left(R_{0}+At^{B}\right)}-ABt^{B-1}\left(ABt^{B-1}-a\right)\left(R_{0}+At^{B}\right)\text{,}$$

to be integrated with the initial condition that h=0 when g(t)=0. The equation has a closed form solution involving the upper incomplete gamma functions and a lengthy algebraic expression, such that it is practically more convenient to solve it numerically. The functional form of h=f(g) can be obtained as a parametric plot of h(t) versus g(t); this in turn allows numerical evaluation of $f^{\prime }\left(g\right)$ and the calculation of the velocity and pressure fields via eqns (2), (3).

Let us now introduce the non-dimensional groups

(7) $$\bar{t}=\frac{at}{R_{0}};\;\bar{h}=\frac{h}{R_{0}^{2}a};\;\dot{\bar{h}}=\frac{d\bar{h}}{d\bar{t}}=\frac{\dot{h}}{R_{0}a^{2}};\;\gamma =AR_{o}^{B-1}a^{-B}\text{;}$$

and note that eq. (6) can be written in non-dimensional form as

(8) $$\dot{\bar{h}}=\bar{h}\frac{\left(\gamma B{\bar{t}}^{B-1}-1\right)}{\left(1+\gamma {\bar{t}}^{B}\right)}-\gamma {\bar{t}}^{B-1}\left(\gamma B\bar{t}-1\right)\left(1+\gamma {\bar{t}}^{B}\right)\text{;}$$

showing that the non-dimensional problem depends exclusively on the parameters γ and B.

For the particular case B=3/2, common in real applications, it was found that the h(t) versus g(t) data is adequately fitted by $f\left(g\right)=c_{1}g\left(e^{-c_{2}g^{2}}+c_{2}g^{2}-1\right)$, where $c_{1}$ and $c_{2}$ are fitting parameters. This results in a pressure field, for R<r<at.

(9) $$p\left(r,t\right)=p_{0}-\frac{\rho a}{r}∙\left(c_{1}∙\left(e^{-c_{2}{\left(r-at\right)}^{2}}\left(1-2c_{2}{\left(r-at\right)}^{2}\right)+3c_{2}{\left(r-at\right)}^{2}-1\right)\;\right)\text{.}$$

Suitable values of $c_{1}$ and $c_{2}$ are given in Table 1 for different values of γ. These were calculated for different case studies considering methane-air and hydrogen-air mixtures, by fitting the expression $f\left(g\right)=c_{1}g\left(e^{-c_{2}g^{2}}+c_{2}g^{2}-1\right)$ to the data measured in Ref. [7].

**Table 1:** Optimal values of fitting coefficients for B=3/2 and different values of γ.

γ	0.000076	0.000241	0.000540	0.002048	0.006475	0.014480
$c_{1}$	$-2.19∙10^{-01}$	$-1.95∙10^{-01}$	$-9.98∙10^{-02}$	$-1.84∙10^{01}$	$-1.61∙10^{01}$	$-7.30∙10^{00}$
$c_{2}$	$2.80∙10^{-05}$	$3.14∙10^{-05}$	$6.23∙10^{-05}$	$2.60∙10^{-03}$	$2.98∙10^{-03}$	$6.65∙10^{-03}$

### 1.2 Benchmark CFD simulations

To test the adequacy of the assumptions made in developing the model we compare the predictions of the model to the results of more detailed CFD simulations, explicitly modelling the ignition and combustion of the gas as well as the consequent propagation of a spherical flame front and radiation of pressure waves. The simulations were conducted in OpenFOAM, using the unsteady Reynolds-averaged Navier Stokes (URANS) technique and the XiFoam solver, developed for modelling compressible premixed combustion with turbulence [11]. The k−ϵ approach was used to model turbulence, where k is the turbulent kinetic energy and ϵ is the rate of dissipation of turbulent kinetic energy, which is calculated as $\epsilon =C_{\mu }^{0.75}k^{1.5}/l$ [11]. Here, $C_{\mu }$ is a coefficient for the turbulent viscosity, set to 0.09, and l is the turbulence length scale. The compressible fluid was modelled as perfect gas with heat capacity ratio specific of the mixture considered (stoichiometric hydrogen-air).

**Fig. 1:**
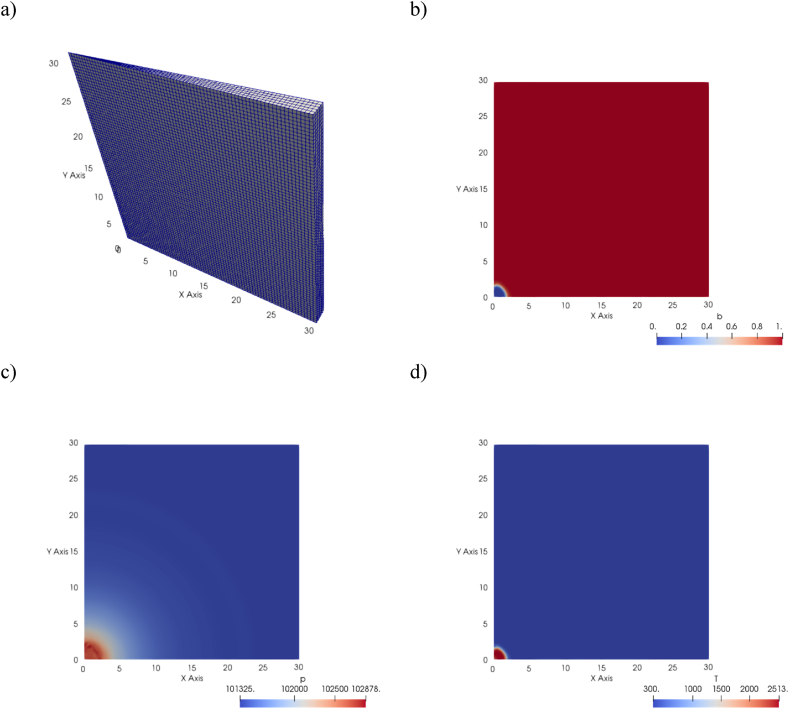
CFD model domain, a) mesh, b) reaction progress variable b, c) pressure p [Pa], d) temperature T [K] at a time of 0.062 s.

A small wedge of an open gas domain was modelled to capture the spherical deflagration event, as sketched in Fig. 1a. The wedge had square faces of length 30 m and maximum thickness of 2 m. Periodic boundary conditions were imposed on the two square faces of the wedge; symmetry boundary conditions were imposed on the plane y=0, while the two remaining surfaces had wave-transmissive boundary conditions, to prevent reflections of the pressure waves at the boundaries of the domain. Ignition was initiated at the origin of the reference system shown in Fig. 1; the cell at that position was initialised to have temperature equal to that of the burning fluid $T_{b}$, i.e. $T=T_{b}$, or $b=\left(T_{b}-T\right)/\left(T_{b}-T_{u}\right)=0$, which defines the reaction progress variable b, with $T_{u}$ being the temperature of the unburned mixture. This variable is initialised to have value of 1 everywhere in the domain except for the ignition region where b=0, and is updated as the simulation progresses.

A static meshing approach was adopted, based on a uniform, structured grid consisting of hexahedral elements in the whole domain, with the exception of triangular prisms adjacent to the tip of the wedge. Two URANS analyses were conducted on this geometry, differing as follows. In all simulations 5 cells were meshing the variable thickness of the wedge, but while in URANS1 the cells were square with size of 0.4 m in the xy plane, this size was 0.2 m in URANS2. In both URANS simulations k was initialised as $1m^{2}/s^{2}$ and the initial value of ϵ was calculated as $\epsilon =C_{\mu }^{0.75}k^{1.5}/l$, where l was set to 10% of the mesh size in the xy plane, taken as the characteristic length of the problem.

**Fig. 2:**
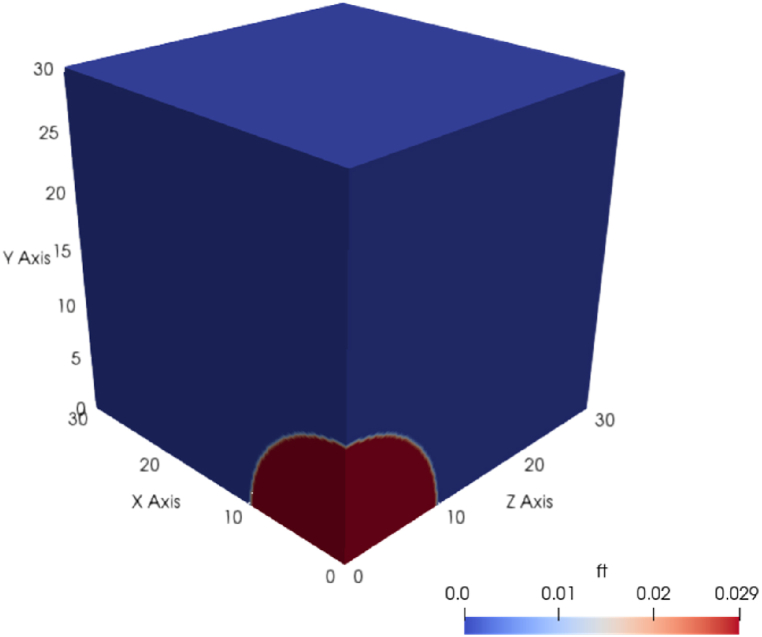
Geometry used in URANS3 and LES and contours of the initial fuel-to-air ratio.

To reassure the reader about the accuracy of the URANS simulations performed in this study, we also conducted a simulation referred to as URANS3, and compared its predictions to those of a LES simulation and to experimental measurements. In URANS3 and LES we model a different domain compared to URANS1 and URANS2, as we try and reproduce the measurements taken in a large-scale controlled hydrogen deflagration experiment [12,13] in which a hemispherical balloon or radius 10 m was filled with a stoichiometric mixture of hydrogen and air before igniting at its centre. In these additional simulations we neglect the presence of the balloon but we model the fact that the flammable mixture is surrounded by atmospheric air. Exploiting the symmetry of the problem, one quarter of the domain was modelled, as shown in Fig. 2; symmetry boundary conditions were imposed on the planes x=0 and z=0; solid wall boundary conditions were imposed on the plane y=0, to capture ground effects, while the three remaining surfaces had wave-transmissive boundary conditions, to prevent reflections of the pressure waves at the boundaries of the domain. Ignition was initiated at the origin of the reference system, coinciding with the center of the hemisphere of flammable gas mixture. Similarly to URANS1 and URANS2, a static meshing approach was adopted, based on a uniform structured grid consisting of hexahedral elements in the whole domain. In the URANS3 and the LES simulation, the mesh size was set to 0.05 and 0.1 m, respectively, obtained from mesh convergence studies. In the LES the time step was set to $10^{-5}$ s, and the one-equation eddy-viscosity SGS model was used to model the Sub-Grid Scale motions [11]. In all URANS it was imposed that the maximum time step of the explicit solver should give a Courant-Friedrichs-Lewy number (CFL) lower than 0.1. Initialization of the turbulence parameters k and ϵ for URANS3 was done as described above for URANS1 and URANS2.

## 2 Results and discussion

**Fig. 3:**
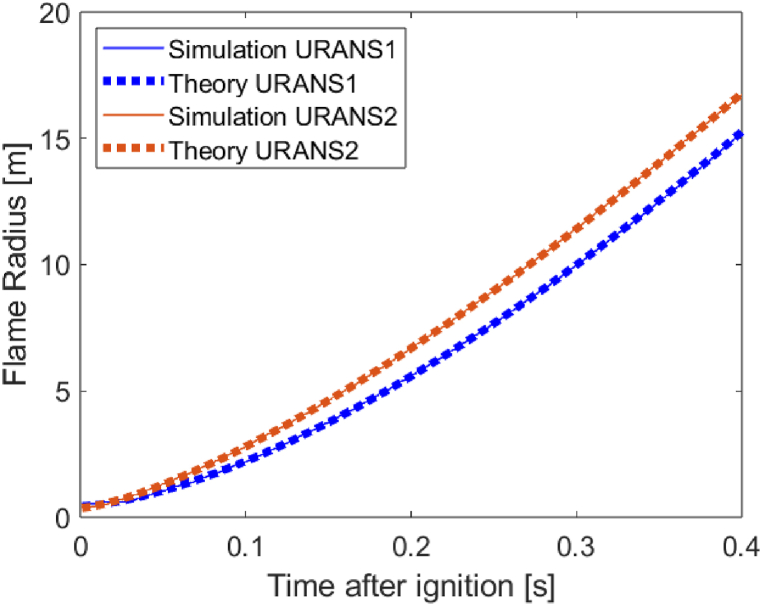
Flame propagation predicted by the CFD simulations, fitted to power-law expressions.

The flame propagation involves a gradual transition of the reaction progress variable b from 1 to 0. Such transition occurs over a finite thickness, which tends to increase with time. Here we extract the position of the flame front from the simulations as the locus of points where b=0.5. The measured flame front propagation in the URANS1 and URANS2 is shown in Fig. 3, which includes power-law fittings of the simulation data.

**Fig. 4:**
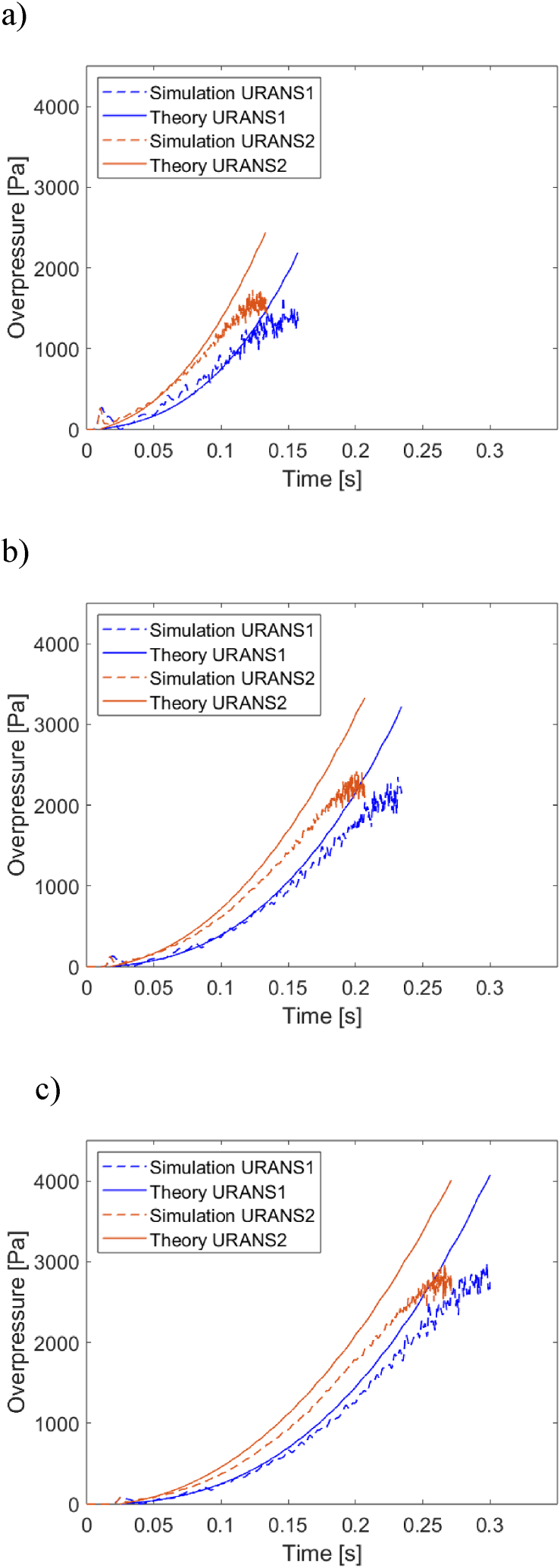
Comparison of the overpressure histories at a) 4 m, b) 7 m, c) 10 m from the ignition point, as predicted by the analytical model or by the CFD simulations.

The two simulations are intending to capture the propagation of an unconfined spherical deflagration front through a homogeneous mixture of flammable gases. The two flame trajectory curves do not coincide as they possess different meshes and different sets of turbulence parameters, but follow closely the expected power-law relation in the range shown. The values of the constants A and B were determined for each of the two curves in Fig. 3 and represented inputs to the analytical model presented above. They were found to be of 60.14 m/s^1.53^, 1.53 and 57.91 m/s^1.38^, 1.38 for URANS1 and URANS2, respectively. The overpressures, calculated via CFD or predicted by the analytical model, are shown in Fig. 4 at different positions. The two sets are initially in excellent agreement and diverge slightly with passing time, as the flame front approaches the measurement point. We note that the analytical predictions are plotted in their validity range, i.e. until the flame front reaches the measuring position $r_{m}$, which occurs at time $t_{m}={\left(\left(r_{m}-R_{0}\right)/A\right)}^{1/B}$.

We recall that the CFD simulations capture the effects of gas compressibility, while the model assumes an incompressible fluid; however, Taylor showed [6] that the effects of compressibility are negligible in the regime explored in this study. We conjecture that the small discrepancy is likely to be related with the finite thickness of the flame front; we note that the overpressure history predicted by the CFD simulation becomes noisier towards the end of the observation, and before the time $t_{m}$ at which the idealised (zero-thickness) flame front reaches the measurement point; this indicates that turbulent combustion is reaching the measurement point earlier than $t_{m}$. In addition we note that the agreement between model and CFD becomes worse as the measuring position $r_{m}$ increases, consistent with the notion that the flame front thickness increases as the flame keeps expanding in space.

We now proceed to apply the proposed analytical model to a large scale deflagration test conducted in 1983 at the Fraunhofer Institut Chemische Technologie (FhG-ICT) [12,13]. This is the largest known deflagration experiment, and measurements taken in this experiment are frequently used as calibration and benchmark for numerical studies [14,15]. In this large scale test a considerable amount of turbulent combustion is observed, which presents a further challenge when making predictions. The test involved the ignition and deflagration of a stoichiometric hydrogen-air mixture contained in a semispherical light polyethylene balloon of radius 10 m. The flame propagation history and overpressure readings at selected distances from the ignition point, inside or outside the balloon, are available in Refs. [12,13]. Before presenting the predictions of the proposed model for this experiment, we make a few caveat: i) the experiment has cylindrical rather than spherical symmetry, due to the presence of the ground and the boundary layer that this induces; ii) the flammable mixture has finite volume, which eventually causes the flame to decelerate and stop; consequently the power-law relation used in this study is only valid initially; iii) the flammable mixture is contained in a structure of non-negligible mass and stiffness, and whether this is equivalent to a situation where the flammable mixture is actually unconfined and surrounded by atmospheric air is debatable [16].

**Fig. 5:**
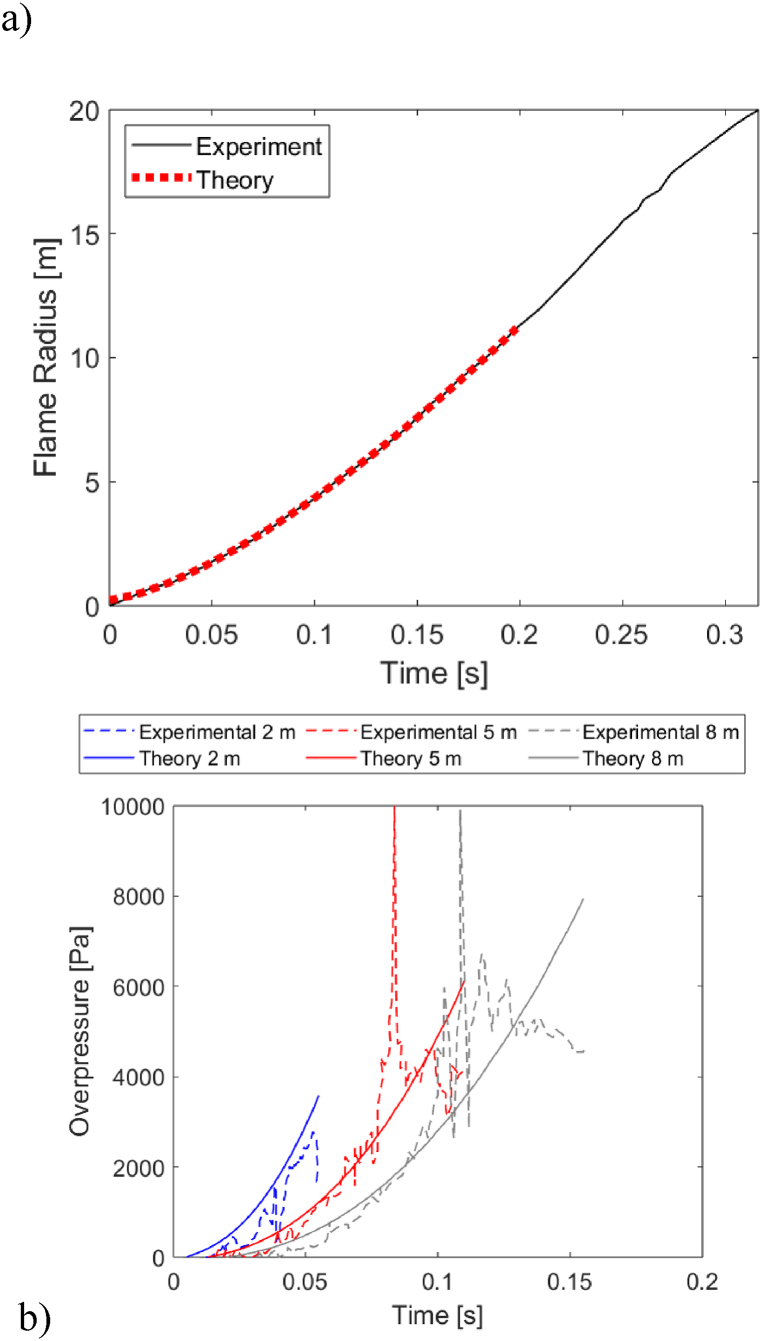
a) Flame propagation history measured in the experiment, fitted to a power-law expressions for *t <* 0.2 s; b) comparison of predicted and measured overpressure histories inside the hydrogen-filled balloon, at different distances from the ignition point.

The measured flame propagation history, obtained from analysis of high-speed footage, is presented in Fig. 5a; an initial portion of this curve was fitted by a power-law, and the power-law parameters ($R_{0}=0.24\;m$, $A=110.5\;m/s^{1.43}$, B=1.43) were determined and used an inputs for our analytical model. In Fig. 5b we present measurements of the overpressure histories at 3 different distances from the ignition point, all inside the balloon, together with the corresponding analytical predictions. We limit our discussion to t<0.2 s, which is the range of validity of the power-law fit in Fig. 5a; this is a sufficiently long time to observe the pressure rising and the flame front beginning to affect the readings at all 3 measurement positions. For clarity, analytical predictions and measurements are plotted up to the $t_{m}$ corresponding to the 3 measurement positions $r_{m}$. It is clear that the proposed model is in good agreement with the measurements, despite the caveats stated above. It captures the arrival time of the wave at the 3 locations and the ensuing rise of the overpressure with time; the time of flame arrival $t_{m}$ is also in broad agreement with the experimental observations. The pressure is captured with an accuracy of circa 10%, excluding some peaks in the pressure measurements. It is noted that a computational time of about 24 h was needed to complete this CFD simulation, on a workstation with 4 CPU cores (Intel® Core™ i7–3770 CPU @3.40 GHz) and 32 GB of RAM. The analytical predictions required a comparably negligible computational time.

**Fig. 6:**
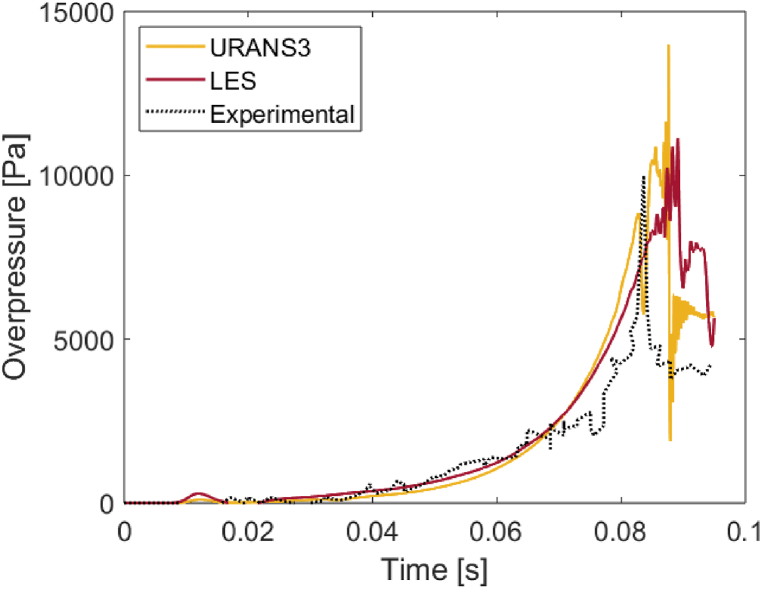
Comparison of the predicted and measured overpressures at 5 m from the ignition point. The results of URANS3 and LES are compared to the overpressure measurements in Refs. [12,13].

In Fig. 6 we compare the overpressure history at 5 m from the ignition point, as measured or predicted by URANS3 and LES. The 3 curves are clearly in good agreement, highlighting two points: i) the less detailed URANS3 simulation used in this study have accuracy comparable to those of the more complete LES, and ii) both simulations agree well with the experimental results. This serves to reassure the reader regarding the accuracy of the URANS simulations presented here.

## 3 Conclusions

We proposed a simple analytical model to predict the pressure radiated by an expanding, unconfined spherical flame front propagating through a premixed flammable mixture, without the need of computationally demanding CFD simulations. The model was shown to be in good agreement with both CFD simulations of a spherical deflagration problem as well as with measurements taken in a real deflagration experiment, in which a hemispherical volume of flammable mixture was confined by a lightweight balloon surrounded by air at atmospheric pressure.

The proposed model can be used to make fast, accurate and inexpensive predictions of the loading history induced by deflagration events on the immediately surrounding structures. In the common case of B≈3/2, an analytical expression is provided for the deflagration pressure wave.

## Data availability

Data can be obtained in the near future from the corresponding authors upon reasonable request.

## Additional information

No additional information is available for this paper.

## CRediT authorship contribution statement

**V. Bisio:** Data curation, Formal analysis, Investigation, Validation, Writing – original draft. **F. Montomoli:** Investigation, Project administration, Supervision. **S. Rossin:** Investigation, Project administration, Supervision. **V.L. Tagarielli:** Conceptualization, Formal analysis, Methodology, Project administration, Supervision, Writing – review & editing.

## Declaration of competing interest

The authors declare that they have no known competing financial interests or personal relationships that could have appeared to influence the work reported in this paper.
